# *Punica granatum L.* Fruit Aqueous Extract Suppresses Reactive Oxygen Species-Mediated p53/p65/miR-145 Expressions followed by Elevated Levels of *irs-1* in Alloxan-Diabetic Rats

**DOI:** 10.22074/cellj.2018.4550

**Published:** 2017-11-04

**Authors:** Ehsan Gharib, Shideh Montasser Kouhsari, Maryam Izad

**Affiliations:** 1 Department of Cellular and Molecular Biology, School of Biology, University College of Science, University of Tehran, Tehran, Iran; 2 Department of Immunology, School of Medicine, Tehran University of Medical Sciences, Tehran, Iran

**Keywords:** miR-145, *p53*, *p65*, *Punica granatum L.*, Reactive Oxygen Species

## Abstract

**Objective:**

Reactive oxygen species (ROS) is an apoptosis inducer in pancreatic β-cells that stimulates p53/p65 mediated microRNA (miR)-145 expression. *Punica granatum L.* (pomegranate) is an antioxidant fruit that attenuates ROS generation. This study examines the effects of pomegranate fruit aqueous extract (PGE) on the levels of ROS, *p53, p65,* miR-145, and its target insulin receptor substrate 1 (irs-1) mRNA in Alloxan-diabetic male Wistar rats.

**Materials and Methods:**

In this experimental study, diabetic rats received different doses of PGE. The effects of the PGE polyphenols were examined through a long-term PGE treatment period model, followed by an evaluation of the plasma and tissue contents of free fatty acids (FFAs), triglycerides (TG), and glycogen compared with diabetic controls (DC)and normal controls (NC). We used real-time polymerase chain reaction (PCR) to investigate the modulation of *p53, p65,* miR-145, and irs-1 expression levels.

**Results:**

There was a noticeable reduction in fasting blood glucose (FBG) and ROS generation compared to DC. We observed marked decreases in *p53, p65,* miR-145 expression levels followed by an elevated level of irs-1, which contributed to improvement in insulin sensitivity.

**Conclusion:**

PGE administration downregulated miR-145 levels in Alloxan-diabetic Wistar rats by suppression of ROS-mediated *p53* and *p65* overexpression.

## Introduction

MicroRNAs (miRNAs) are small, non-coding RNA molecules that play a central role in the regulatory network of metabolic pathways (1). miR-145 is a member of the miR-143-145 cluster and acts as a negative regulator of insulin receptor substrate 1 (IRS-1) level by pairing with the 3’-UTR region of *irs-1* mRNA (2). Researchers report upregulation of miR-145 in obesity and increased lipolysis in adipocytes (3). As a result, miR-145 stimulates hyperglycemia and hyperlipidemia due to insulin signaling pathway disruption (4). Also, a recent study has reported miR-145 upregulation in type 1 diabetes mellitus (T1DM) patients and an animal model (5).

The miR-145 expression level is associated with reactive oxygen species (ROS) accumulation. Destruction of insulinsecreting pancreatic β-cells in T1DM results in massive ROS generation in response to mitochondrial defection (6). An enhanced level of ROS content upregulates the p53 and p65 subunit of the transcription factor nuclear factor-κB (NF-κB) complex expressions and transcriptional activities (7, 8), which have a positive impact on miR-145 overexpression (8, 9). ROS is identified as a positive regulator of inflammatory cytokines such as tumor necrosis factor-alpha (TNF-α); therefore, it stimulates p65 induction of miR-145 expression in an alternative way (9, 10).

*Punica granatum L.* (pomegranate) is a widely used plant and appears to be native in some parts of Asia, particularly Iran. Extract of the pomegranate fruit has a high polyphenolic content that includes punicalagin, anthocyanins, ellagic acid, gallic acid, caffeic acid, catechins, quercetin, and rutin (11). The pomegranate is known as an antioxidant-rich fruit because of its positive effects in limiting oxidative stress in humans (12) and rats (13). Based on the main contribution of the liver and skeletal muscle tissues in blood glucose homeostasis (14), downregulation of miR-145 could be considered a therapeutic method in obesity, metabolic syndrome, and T1DM subjects. Due to the negative effects of the pomegranate on ROS production, inflammation, p53 and p65 levels (15-17), we hypothesized that treatment with the pomegranate fruit aqueous extract (PGE) could ameliorate miR-145 levels in alloxan-induced diabetic rats (as an animal model for T1DM) via downregulation of p53/p65 expressions. Therefore, we analyzed the expression levels of p53, p65, and miR-145, along with the mRNA content of *irs-1* in diabetic rats treated with different doses of PGE compared to diabetic and healthy controls

## Materials and Methods

In this experimental study, chemicals and reagents were obtained from the following sources: Alloxan monohydrate (Sigma, St. Louis, MO, USA), RNA Extraction kit (SinaClon Co., Iran), PrimeScript RT Reagent kit (TaKaRa Biotechnology, Japan), miRNA isolation kit and Super SYBR Green qPCR Master Mix 2x (Yekta Tajhiz Co., Iran), Universal cDNA synthesis kit II and ExiLENT SYBR® Green PCR Master Mix (Exiqon, USA), Rat Reactive Oxygen Species ELISA kit (MyBioSource, San Diego, CA, USA), Triglyceride Quantification Assay kit (Abcam, USA), and commercial Free Fatty Acid Assay kit (Enzychrom Bio-Assays Systems, USA). All other chemicals and solvents were of the highest commercial grade and purchased from either Merck (KGaA, Germany) or Sigma (St. Louis, MO, USA). We purchased male Wistar rats (Rattus norvegicus) that weighed 180- 220 g from the School of Pharmacy, Tehran University of Medical Sciences (Iran). Animals were housed 4 per standard rat cage in a room with a 12:12 hour light/dark cycle and controlled temperature (24 ± 1˚C). The animals were allowed to adapt to their new location for one week. Animals received water and a standard diet (27% protein, 32% fat, and 41% carbohydrate) ad libitum.

### Ethical consideration

All procedures that involved animals and their care that included feeding, extract administration, blood sampling, gastric intubation, anesthesia, and euthanasia were under the close supervision of qualified and experienced personnel, and approved by the Institutional Animal Care and Use Committee of University of Tehran.

### Preparation of the pomegranate fruit aqueous extract

Pomegranate fruits were washed and manually peeled without separating the seeds. The extract was obtained using a blender, filtered through Whatman No. 1 filter paper to remove any water insoluble materials, and then steamed (5 minutes) for enzyme inactivation. In the next step, the extract was stored at -18˚C. The extract subsequently underwent a drying process in a cabinet dryer at 37˚C for 72 hours and was suspended in distilled water at doses of 100, 200, and 350 mg/kg body weight (bw).

### Preparation of the alloxan-induced diabetic Wistar rats

Diabetes was induced in rats that fasted for 18 hours by a single intraperitoneal injection of alloxan monohydrate (120 mg/kg) freshly dissolved in cold citrate buffer (pH=4.5) subcutaneously. At 72 hours following the injection, we confirmed hyperglycemia in the rats via blood samples from the tail vein which were measured by a glucometer device (On Call Now, San Diego, CA, USA). The animals were considered diabetic if their blood glucose levels were 300 mg/dl or higher for two consecutive measurements.

### Experimental design

Rats were randomly divided into 8 groups of 12 rats per group and placed in cages (two rats per cage). They were treated orally with PGE for a period of 21 days as follows: group I normal control (NC): normal rats treated with vehicle alone. Group II (PGE+Na): normal rats treated with PGE at a dose of 100 mg/kg bw. Group III (PGE+Nb): normal rats treated with PGE at a dose of 200 mg/kg bw. Group IV (PGE+Nc): normal rats treated with PGE at a dose of 350 mg/kg bw. Group V diabetic control (DC): diabetic rats treated with vehicle alone. Group VI (PGE+Da): diabetic rats treated with PGE at a dose of 100 mg/kg bw. Group VII (PGE+Db): diabetic rats treated with PGE at a dose of 200 mg/kg bw. Group VIII (PGE+Dc): diabetic rats treated with PGE at a dose of 350 mg/kg bw. These doses were selected based on results obtained in pilot experiments that used lower doses of PGF (10 and 50 mg/kg/day). These doses failed to ameliorate hyperglycemia and other clinical outcomes (data not shown), whereas the selected doses were found to be efficient. At the end of the experimental period, the rats fasted overnight and we obtained blood samples via intra-cardiac puncture (under anesthesia) on day 21. Blood samples were collected in both ordinary and lithium heparin tubes. For serum separation, blood samples were incubated at room temperature to allow for clotting, and then centrifuged at 1000 g for 15 minutes for plasma separation from the erythrocytes. Sera were stored in aliquots at -70˚C until biochemical analysis. Liver and skeletal muscle tissues were collected, weighed, and frozen in liquid nitrogen, and stored at -180˚C.

### Biochemical analysis

We assessed β-cell function in the experimental groups by measuring serum insulin levels by the ELISA method (Demeditec, Germany) according to the manufacturers’ instructions. The plasma TG and free fatty acid (FFA) analyses were performed on the samples, which were collected at the end of the experimental period (day 21). Samples were then quantified using Triglyceride and Free Fatty Acid Quantification Assay kits, according to the manufacturers’ instructions. To estimate the effect of PGE administration on the TG level of the liver and skeletal muscle tissues, portions of the samples obtained from the control and treated groups were suspended and homogenized in 1 ml of 5% NP-40/ddH_2_O, boiled, and later centrifuged at 12000 g for 2 minutes. The tissue TG concentrations were analyzed using the same kit as described for the plasma TG analysis. ELISA assay was performed to assess the ROS levels in the plasma, liver, and skeletal muscle tissues of the experimental groups. Collected samples were prepared and subjected to quantitative analysis with a Rat Reactive Oxygen Species ELISA kit according to the manufacturers’ protocol. To determine the glycogen content in the liver and skeletal muscle, tissue samples were dissolved in 30% KOH, boiled, and later centrifuged at 12000 g for 5 minutes. Glycogen was measured in the supernatants with a commercial glycogen assay kit.

### RNA extraction and real-time polymerase chain reaction analysis

Total RNA was extracted from the homogenized suspension of the target tissues using a Polytron PT1600E bench-top homogenizer (Kinematica AG, Switzerland) and RNX-Plus reagent kit according to the manufacturers’ instructions. The RNA concentration was measured by a Nanodrop ND- 1000 spectrophotometer (Nanodrop Technologies), and the quality was assayed by measuring the A260/A280 ratio of 1.8-2.0. A total of 1 μg total RNA was reverse-transcribed with the PrimeScript RT reagent kit. Quantitative real-time reverse transcription-PCR was carried out on an ABI Step One RT-PCR thermal cycler (ABI Stepone, NY, USA) using a YTA SYBR green qPCR masterMix 2X kit according to the manufacturers’ instructions. Primer sequences were designed for all genes using PerlPrimer. Primer-BLAST (NCBI) was then used to check their specificity. Ribosomal 18S RNA was utilized to calculate the relative abundance of the mRNA transcripts. We assessed RNA integrity by measuring ribosomal 28S RNA levels. Each measurement was performed in triplicate. The primer sequences for realtime PCR were as follows:

p53- F: 5ˊ-CTACTAAGGTCGTGAGACGCTGCC-3ˊ
  R: 5ˊ-TCAGCATACAGGTTTCCTTCCACC-3ˊ
p65- F: 5ˊ-CTTCTGGGCCATATGTGGAGA-3ˊ
  R: 5ˊ-TCGCACTTGTAACGGAAACG-3ˊ
irs-1- F: 5ˊ-GATACCGATGGCTTCTCAGACG-3ˊ
  R: 5ˊ-TCGTTCTCATAATACTCCAGGCG-3ˊ
18S F:5ˊ-GGACACGGACAGGATTGACA-3ˊ
  R: 5ˊ-ACCCACGGAATCGAGAAAGA-3ˊ
28S F:5ˊ-GGTAAACGGCGGGAGTAACTATG-3ˊ
  R: 5ˊ-TAGGTAGGGACAGTGGGAATCTCG-3ˊ
miR-145: 5ˊ- GUCCAGUUUUCCCAGGAAUCCCU-3ˊ

### MicroRNA extraction and real-time-polymerase chain reaction analysis

The miRNAs were extracted from the plasma and homogenized suspension of liver and skeletal muscle tissues using the miRNA isolation kit according to the manufacturers’ instructions. The concentration of purified RNA was determined in the Nanodrop ND- 1000 spectrophotometer and quality was estimated by A260/A280 ratio of 1.8-2.0. We added the UniSp6 RNA Spike-in template to each sample. A total of 1 μg of the miRNA samples was reverse-transcribed with the Universal cDNA synthesis kit II. Quantitative real-time reverse transcription-polymerase chain reaction (PCR) was carried out on an ABI Step One RT-PCR thermal cycler, using a specific miR-145 LNA™ primer (Exiqon, USA) and ExiLENT SYBR® Green PCR Master Mix that contained the miScript Universal reverse primer, according to the manufacturers’ instructions. Relative miRNA abundance was determined by normalization to U6 using the 2^-ΔΔCT^ method.

### Statistical analysis

All data are mean ± SD. Comparisons between groups were made by one-way analysis of variance (ANOVA) followed by an appropriate post-hoc test to analyze the difference. Statistical significance was achieved at P<0.05.

## Results

### Pomegranate fruit aqueous extract increases the insulin level in diabetic rats

**Fig.1:**
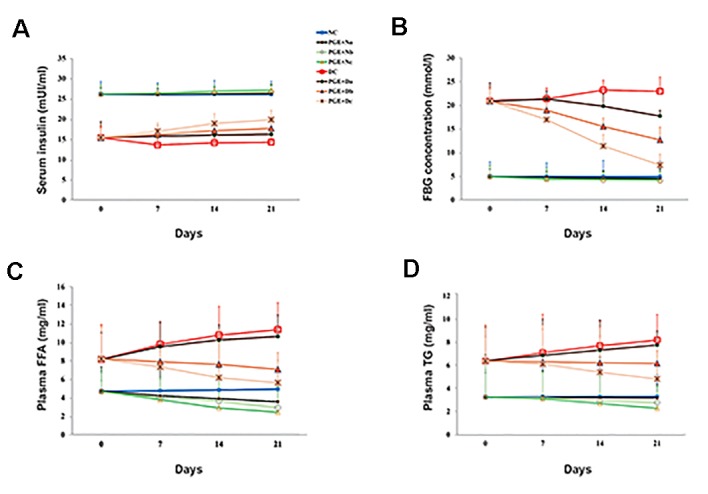
Biochemical analysis in the experimental rats. Effects of pomegranate fruit aqueous extract (PGE) on A. Insulin, B. Fasting blood glucose, C. Free fatty acid (FFA), and D. Triglyceride (TG) concentrations were evaluated in the non-diabetic control group (NC, n=12), non-diabetic group treated with 100, 200, or 350 mg/kg body weight (bw) of pomegranate fruit aqueous extract [PGE+N (a, b, c); n=12], diabetic control group (DC, n=12), and diabetic group treated with 100, 200, or 350 mg/kg bw of PGE [PGE+D (a, b, c); n=12]. Each value is the mean ± SD of six separate experiments.

In comparison with the control group, we observed decreased serum insulin levels in the DC rats (Fig .1A). In contrast to DC, the daily PGE intake elevated insulin levels in the diabetic groups, as with PGE+Dc, the data were statistically significant (P<0.001). SPSS software version 20 (SPSS Inc, USA) was used in this study.

### The impact of pomegranate fruit aqueous extract consummation on hyperglycemia

We measured glucose concentrations were measured in the target groups after 7, 14, and 21 days (Fig .1B). In DC rats, fasting blood glucose (FBG) values increased significantly. The proportion of FBG in the PGE treated animals, however, reduced compared to the first day and DC controls (P<0.001).

### Pomegranate fruit aqueous extract improves hyperlipidemia in target groups

Prolonged PGE treatment showed a promising reduction in plasma FFA and TG concentrations compared to the DC group (Fig .1C, D). The PGE+Dc rats, however, had significant differences (P<0.001).

### Administration of pomegranate fruit aqueous extract reduces reactive oxygen species level in alloxandiabetic rats

**Fig.2:**
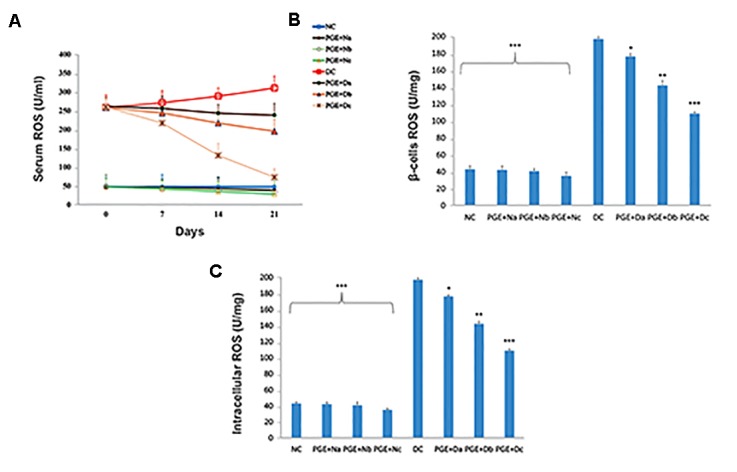
The evaluation of the reactive oxygen species (ROS) contents. Analysis were performed on A. Serum, B. β-cells, and C. Intracellular of alloxan-diabetic rats. NC; Normal control (n=12), DC; Diabetic control (n=12), pomegranate fruit aqueous extract (PGE)+N (a, b, c); Normal rats treated with 100, 200, or 350 mg/kg body weight (bw) of PGE (n=12), PGE+D (a, b, c); Diabetic rats treated with 100, 200, or 350 mg/kg bw of PGE (n=12), Significantly different from DC group, *; P<0.05, **; P<0.01, and ***; P<0.001. Each value is the mean ± SD of six separate experiments.

We determined serum ROS concentrations in the experimental groups during a 3-week treatment period with PGE. When compared with the healthy controls, DC rats showed additive ROS levels during diabetes progression (Fig .2A). This process reversed in the PGE treated groups and significantly reduced in the PGE+Dc rats (P<0.001). Pancreatic ROS levels were assessed to determine the effect of prolonged treatment on β islets (Fig .2B), and liver and skeletal muscle tissues (Fig .2C). As expected, ROS accumulation significantly decreased in PGE administered diabetic rats compared to DC controls (P<0.001). Interestingly, normal rats that received the extract also exhibited smaller amounts of ROS production in their serum, as well as in the pancreas, liver and skeletal muscle tissues.

### Pomegranate fruit aqueous extract ameliorates *p53* and *p65* overexpression

**Fig.3:**
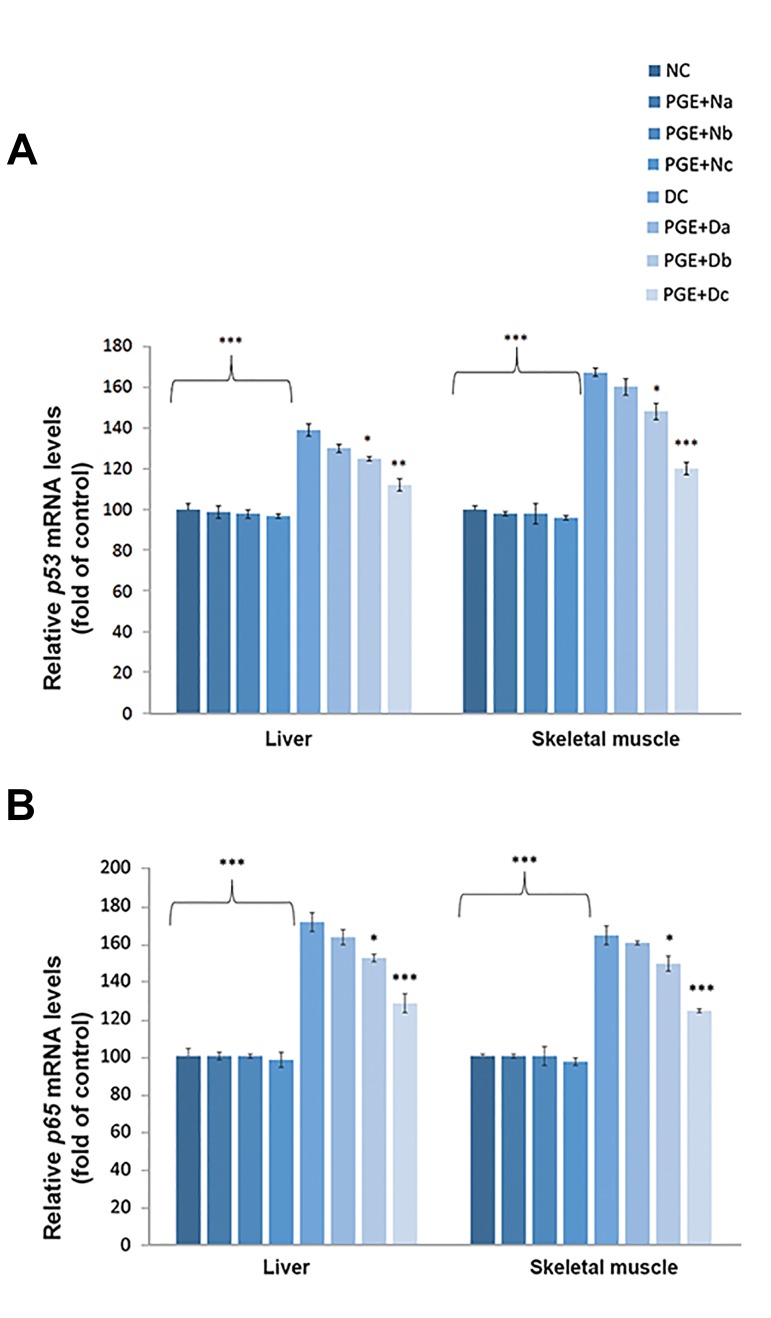
Quantitative real-time polymerase chain reaction analysis of the inflammatory transcription factors. The levels of the A. *p53* and B. *p65* were measured in the liver and skeletal muscle tissues of non-diabetic control group (NC, n=12), non-diabetic group treated with 100, 200, or 350 mg/kg body weight (bw) of pomegranate fruit aqueous extract [PGE+N (a, b, c); n=12], diabetic control group (DC, n=12), and diabetic group treated with 100, 200, or 350 mg/kg bw of PGE [PGE+D (a, b, c), n=12]. Results are expressed as fold of control. 18s RNA was used as an internal control (n=3). The mean of six independent experiments is shown. Significantly different from DC group, *; P<0.05, **; P<0.01, and ***; P<0.001.

In the alloxan diabetic rats, *p53* expression upregulated in response to a high level of ROS production, which significantly differed from those in the NC group (Fig .3A). Oral PGE administration (350 mg/kg bw) resulted in a marked reduction of the *p53* expression level in the diabetic rats (P<0.01). The level of *p65* expression, as a subunit of the transcription factor NF-κB complex, increased with diabetes progression (Fig .3B). The PGE+Dc treated group had a lower level of p65 (P<0.001), which closely approximated the healthy controls.

### Pomegranate fruit aqueous extract downregulates miR-145 expression in diabetic rats

**Fig.4:**
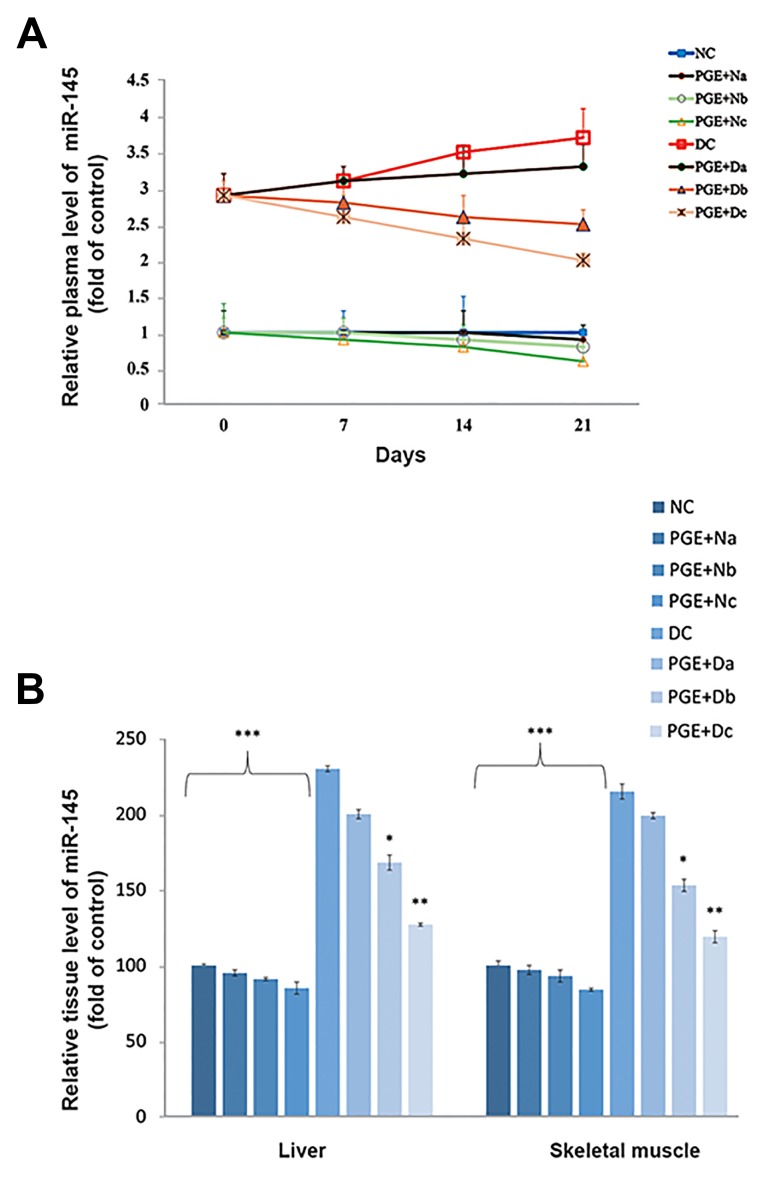
Quantitative real-time polymerase chain reaction analysis of the miR-145 expression levels. The examinations were performed on A. Plasma, B. Liver and skeletal muscle tissues of non-diabetic control group (NC, n=12), non-diabetic group treated with 100, 200, or 350 mg/kg body weight (bw) of pomegranate fruit aqueous extract [PGE+N (a, b, c); n=12], diabetic control group (DC, n=12), and diabetic group treated with 100, 200, or 350 mg/kg bw of PGE [PGE+D (a, b, c); n=12]. Results are expressed as fold of control. U6 was used as an endogenous control (n=3). The mean of six independent experiments is shown. Significantly different from DC group, *; P<0.05, **; P<0.01, and ***; P<0.001.

In comparison to the control group, miR-145 expression upregulated in the plasma (3.7-fold, Fig .4A), and liver (2.3-fold) and skeletal muscle (2.1- fold) tissues (Fig .4B) of DC rats (P<0.001). Of note, the values obtained for the PGE+Dc rats were similar to the NC group.

### Effect of pomegranate fruit aqueous extract treatment on *irs-1* level in target rats

**Fig.5:**
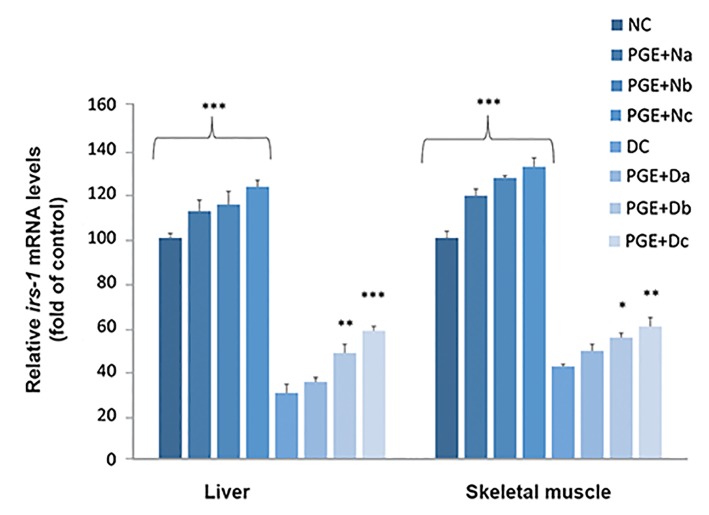
Quantitative real-time polymerase chain reaction analysis of the insulin receptor substrate 1 (irs-1) expression levels. The tests were carried out on the liver and skeletal muscle tissues of the non-diabetic control group (NC, n=12), non-diabetic group treated with 100, 200, or 350 mg/kg body weight of pomegranate fruit aqueous extract [PGE+N (a, b, c); n=12], diabetic control group (DC, n=12), and diabetic group treated with 100, 200, or 350 mg/kg bw of PGE [PGE+D (a, b, c); n=12]. Results are expressed as fold of control. 18s RNA was used as an internal control (n=3). The mean of six independent experiments is shown. Significantly different from DC group, *; P<0.05, **; P<0.01, and ***; P<0.001.

The transcription levels of *irs-1* were examined in the NC, DC, and PGE-treated groups (Fig .5). *irs-1* expression significantly reduced in the DC group. In contrast, irs-1 expression increased in the PGE+Db and PGE+Dc rats.

### Impact of the pomegranate fruit aqueous extract on triglyceride and glycogen contents in liver and skeletal muscle tissues

**Fig.6:**
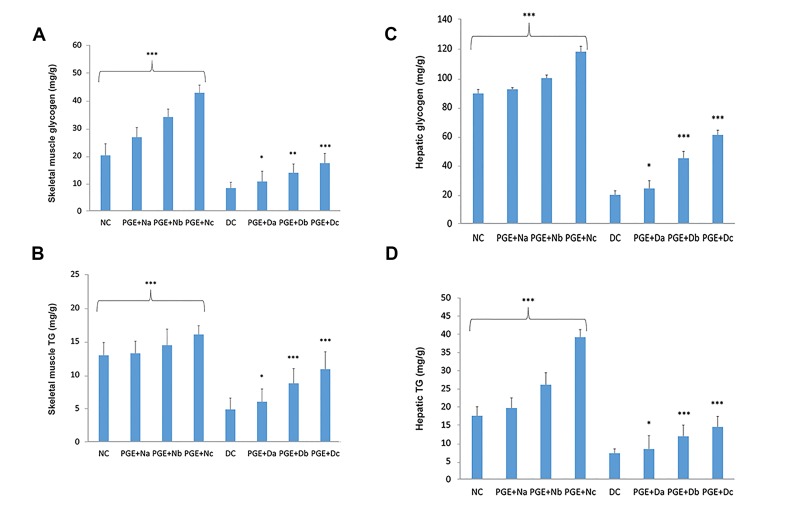
Effects of pomegranate fruit aqueous extract (PGE) on the glycogen and triglyceride (TG) contents. The examinations were performed on the A, B. Liver and skeletal muscle, C. and D. Tissues of the normal control group (NC, n=12), non-diabetic group treated with 100, 200, or 350 mg/kg body weight (bw) of PGE [PGE+N (a, b, c); n=12], diabetic control group (DC, n=12), and diabetic group treated with 100, 200, or 350 mg/kg bw of PGE [PGE+D (a, b, c); n=12]. Each value is the mean ± SD of six separate experiments. Significantly different from DC group, *; P<0.05, **; P<0.01, and ***; P<0.001.

The DC group had the lowest levels of TG and glycogen storages (P<0.001, Fig .6). In the PGE group, uptake increased the TG levels and glycogen storages in diabetic rats close to the levels of the NC group.

## Discussion

In T1DM, immune system cells (T cells and macrophages) act against β-cells by secreting proinflammatory cytokines TNF-α, nitric oxide and ROS, which eventually induce the destruction of β-pancreatic islets (insulitis) (6). Thus, depletions in β-cells lead to decreased insulin secretion and concentration in the body, hyperglycemia, and hyperlipidemia. Elevations in blood glucose have been proven to be strong stimulators of ROS generation in diabetic models (18). For *in vivo* studies, ROSmediated β-cell destruction in rats is established by an injection of alloxan monohydrate. This compound is an oxygenated pyrimidine derivative exclusively taken up by Glut-2 transporters. Alloxan and its reductive product, dialuric acid, create a redox cycle which generates abundant superoxide radicals. Eventually, due to the low levels of antioxidant enzymes, such as catalase, ROS molecules accumulate in β-cells, resulting in inflammation and apoptosis (19) .

p53 is one of the apoptotic mediator proteins that activates in response to ROS elevation and plays a transcription factor role in recruiting the expression of genes involved in cell death or survival (19). Previous investigations have reported an elevated level of *p53* expression according to oxidative stress in alloxan-induced diabetic rats (20). A p53 response element is located in the promoter region of miR-145, which upregulates its expression (8) and may be involved in miR-145 activation during oxidative stress situations (21). According to these evidences, we have hypothesized that ROS accumulation in alloxan-diabetic rats accelerates hyperglycemia and hyperlipidemia through the enhancement of miR-145 expression levels.

Our experiments demonstrated an accelerated level of *p53* gene expression in the liver and skeletal muscle tissues of diabetic rats, in response to massive ROS production followed by high miR-145 expression in target tissues, as well as plasma. IRS-1 is a vital mediator in the insulin metabolic signaling pathway downregulated by miR-145. During the postprandial phase, insulin hormone actives the IRS-1/phosphatidylinositol 3-kinase/protein kinase B (IRS-1/PI3K/Akt) cascade and upregulates glucose uptake and storage (22), lipid synthesis (23) and attenuates lipolysis (24) in cells.

Previous studies have explored the effects of the polyphenols on regulation of miRNAs in cancer (25, 26); however, to our knowledge, there is scant evidence regarding their effects on metabolic complications (27). In this study, the DC group has depicted a decreased level of *irs-1* transcription in response to miR-145 overexpression. In contrast, diabetic rats treated with PGE for 21 days reversed miR-145 elevation with increased *irs-1* mRNA expression. The same results were obtained from healthy treated groups, as oral administration of PGE lowered the basal level of miR-145 expression in all groups and, conversely, elevated irs-1 levels in the healthy animals. These data supported clinical findings that showed PGE consumption improved TG and glycogen storage in normal groups, as well as diabetic rats.

There is a synergy between miR-145 overexpression, high levels of lipolysis, TNF-α secretion, and NF-kB activation. In the alloxan-diabetic rats, in response to ROS accumulation, the NF-kB complex upregulated miR- 145 by binding its p65 subunit to the miR-145 promoter (9). miR-145 inhibitory effects on the insulin signaling pathway have been shown to lead to hydrolysis of TG and FFA release (3). Saturated FFA binds to the macrophage toll-like receptor 4 (TLR4) and stimulates TNF-α secretion. TNF-α provokes mitogen-activated protein kinases (MAPKs) and NF-kB activation by attaching to TNF-α receptor-1 (TNFR-1). This removes the inhibitory effect of perilipin-1 (PLIN1) on the hormone-sensitive lipase (HSL) by downregulation of the PLIN1 expression and acceleration of its phosphorylation, thus intensify TG hydrolysis (3). In line with these reports, our results have revealed that PGE treatment contributed to the improvement in hyperlipidemia as a result of a reduction in ROS-mediated p65 induction of miR-145 expression in alloxan-diabetic rats.

## Conclusion

Overall, the present study showed that oral administration of PGE ameliorated induced hyperglycemia and hyperlipidemia in alloxan-diabetic rats. With regards to the formation of an ROS wave in the rats’ pancreatic β-cells attributed to the alloxan injection, the reductive effect of pomegranate on ROS accumulation improved β-cell function and insulin secretion. The PGE components had a suppressive impact on high *p53* and *p65* expression levels in the liver and skeletal muscle tissues of diabetic rats and, accordingly, implicated miR-145 upregulation by p53/p65 stimulation. Consequently, the decreased irs-1 mRNA level, as a direct target of miR-145, improved in the intended tissues followed by enhanced glucose uptake and storage. However, due to the presence of various active components such as flavonoids in PGE (as described earlier), further studies should be carried out to obtain a deeper understanding of the underlying mechanisms.
